# The analysis of aerobics intelligent fitness system for neurorobotics based on big data and machine learning

**DOI:** 10.1016/j.heliyon.2024.e33191

**Published:** 2024-06-18

**Authors:** Yuanxin Liu, Shufang Cao

**Affiliations:** a Sports Department, Henan Medical College, Zhengzhou, 451191, China; b Ministry of Basic Medicine Education, Dazhou Vocational College of Chinese Medicine, Dazhou, 635000, China

**Keywords:** Neurorobotics, Aerobics, Spark, Big data, Machine learning

## Abstract

In modern society, people's pace of life is fast, and the pressure is enormous, leading to increasingly prominent issues such as obesity and sub-health. Traditional fitness methods cannot meet people's needs to a certain extent. Therefore, this work aims to use technology to change people's lifestyles and compensate for traditional fitness methods' shortcomings. Firstly, this work overviews neurorobotics, providing neural perception and control functions for aerobics intelligent fitness system. Secondly, the connection between big data and machine learning (ML), big data technology products, and the ML process are discussed. The Spark big data platform builds node data for calculation, and the decision tree algorithm is used for data preprocessing. These are important for future intelligent fitness analysis. This work proposes an aerobics intelligent fitness system based on neurorobotics technology and big data analysis and develops a recommendation system for the best fitness exercise. This system utilizes neural perception and control functions, combined with big data and ML technology, to solve the obesity and sub-health problems faced by people in fast-paced and high-pressure lifestyles. By harnessing the computational capabilities of the Spark big data platform and applying the decision tree algorithm for data preprocessing, the system can furnish users with personalized fitness plans and optimization recommendations. This work conducts a model performance study on 35 % aerobic fitness data on intelligent fitness Android v1.0.8 to evaluate the system's data processing ability and training effectiveness. Moreover, the aerobics intelligent fitness system models based on neurorobotics, big data, and ML are evaluated. The results indicate that normalizing the data using the Min-Max method leads to a decrease in the F1 value and a reduction in data set errors. Consequently, the dataset studied by the system model is beneficial to improving the work efficiency of the aerobics intelligent fitness system. After the comprehensive human quality of the system model is evaluated, the actual average score of the comprehensive human quality of the 13 users tested before the aerobics intelligent fitness system test is 91.44, and the average prediction score is 90.88. The results of the two tests are similar. Thus, using the intelligent fitness system can enable the user to obtain system feedback according to the actual training effect, thereby playing a guiding role in the intelligent fitness of aerobics for the user. This work designs and implements the aerobics intelligent fitness system close to the human body's training effect, further enhancing the specialization and individualization of sports and fitness.

## 1 Introduction

With the continuous improvement of people's living standards, the practical value of aerobics for health care, medical treatment, fitness, bodybuilding, and entertainment has been valued by people [1]. This appeal has attracted enthusiasts of various age groups, forming a substantial consumer demographic [2]. Television stations have produced special programs about the competition and popularization of aerobics, and their ratings far surpass other programs [3]. Furthermore, venues for aerobics competitions, such as gymnasiums and stages, experience heavy utilization, providing opportunities for companies to advertise during these events. Consequently, many businesses are interested in sponsoring aerobics-related activities [4]. According to the “Report on Nutrition and Chronic Disease Status of Chinese Residents (2020)” released by the National Health and Medical Commission in 2020, the obesity rate of adult residents, children aged six to seventeen, and children under the age of six in China reached about 50 %, 20 %, and 10 %, respectively. Six hundred million people are overweight and obese, ranking first in the world. The public's health awareness has increased, and the demand for self-actualization fitness, such as muscle building and shaping, is expanding daily [5]. Moreover, with the rise of China's middle class, per capita disposable income and consumption expenditure continue to grow, and consumption in personal care, fitness, and maintenance will become a new trend [6]. The “National Fitness Plan (2021–2025)” proposed that the proportion of people who regularly participate in physical exercise will reach 38.5 % (37.2 % in 2020), and the total scale of the national sports industry will reach the goal of 5 trillion yuan by 2025 [7]. Aerobics is a popular sport that combines group gymnastics, dance, music, fitness, and entertainment [8]. Aerobics absorbs the upper and lower body, trunk, neck, and foot movements in disco, jazz, and break dancing, especially the hip movements, which add vitality to aerobics. It also helps reduce fat accumulation in the buttocks and abdomen. Besides, it improves motor coordination and flexibility [9]. It is necessary to scientifically arrange the exercise time and frequency and pay attention to exercise hygiene to achieve the ideal exercise effect in aerobics [10]. However, fitness often only focuses on physical exercise, neglecting interaction and coordination with the brain. The development of neurorobotics technology has provided new possibilities for intelligent fitness. This work combines neurorobotics technology with big data and machine learning (ML) to create an intelligent aerobic fitness system that solves the obesity and sub-health problems faced by people in modern society through neural perception and control functions and data analysis. With the progress of the Internet of Things (IoT) technology and large-scale sports events, relying on intelligent equipment such as neurorobotics, urban sports are gradually transforming into technology-based intelligence. In the intelligent process of the sports and fitness industry, it is necessary to expand the application scenarios of intelligent fitness further, continuously improve its service experience, and solve the shortcomings of insufficient pertinence of traditional fitness methods. In addition, the main advantage of artificial neurorobotics lies in its dexterous structure and device, which can improve the effect of fitness and aerobic exercise through a friendly human-computer interaction interface.

In the 1980s, modern aerobics rose rapidly. NASA's M.D., Coper, specially designed some aerobic physical training movements for astronauts, accompanied by music and sports clothing, forming a unique sports system that soon swept the world [11]. The famous Hollywood star Jane Fonda's book “Jane Fonda Aerobics” quickly sold in more than 20 countries, and a worldwide aerobics craze began to emerge [12]. Many Internet sports and fitness fields have not yet seen Initial public offerings (IPO) cases. Still, after nearly a decade of development, many financing companies have B rounds and above [13]. Different products compete with each other, and product forms are gradually enriched, but the commercialization of many fitness apps is still to be verified [14]. IBM uses ML and data mining technology to combine sports and big data application technology to extract data generated during sports practice [15]. With the rise of big data technology and information, users have high requirements for sports and fitness products' content and service experience. In the future, under the new demands of the public for fitness, how to innovate products and solve pain points to meet the unique needs of users will become a vital issue for fitness companies [16].

This work proposes an idea of the aerobics intelligent fitness system grounded on neurorobotics to achieve this goal. This system is based on neural perception and control functions, utilizing big data analysis and ML technology to generate intelligent fitness recommendations and personalized training plans through deep learning (DL) models. DL models can learn and identify users' exercise preferences, physical states, and fitness needs from massive data, thereby customizing each user's best exercise plan. This DL model adopts an advanced neural network architecture and achieves precise prediction and analysis of motion data through multi-level feature extraction and learning. By inputting a large amount of aerobic fitness data into the model for training, the system can continuously optimize its recommendation ability and provide users with accurate and personalized fitness guidance. In addition, previously used DL models and related studies of the aerobics intelligent fitness system of neurorobotics were analyzed. Beltrame et al. (2018) [17] used wearable sensors and ML models to extract aerobic capacity system dynamics from unsupervised daily activities. The study aimed to enable the monitoring and evaluation of aerobic capacity in daily activities. Shahin et al. (2020) [18] proposed an accurate and rapid cardiovascular view classification system based on fusion depth features and the Long Short-Term Memory (LSTM) model. The purpose was to apply DL to classify and analyze cardiovascular data. Farrokhi et al. (2021) [19] studied the application of IoT and artificial intelligence (AI) in smart fitness. The research explored the applications and prospects of IoT and AI in smart fitness equipment, data analysis, and personalized training. Li et al. (2022) [20] introduced a physical education guidance model and an AI system for physical fitness assessment based on DL. The research used DL models to realize intelligent assistance to physical education and physical fitness assessment. Sujith et al. (2022) [21] believed that big data and ML can be applied in data security, data privacy, and health detection to achieve customized health goals and healthy lifestyles. Zhang et al. (2023) [22] introduced and embedded attention mechanisms into decoding networks through a universal human body analysis model, constructed spatial graph convolutions, and extended them to the time series dynamics of mobile human bone data. This can effectively monitor rope-skipping data and be used to operate rope-skipping. Patalas-Maliszewska et al. (2023) [23] used inertial sensors and ML algorithm to implement physical exercise correctly and proposed a Convolutional Neural Network (CNN) with additional post-processing blocks to recognize sports scenes. The results showed that the recognition rate of this method for sports events was 0.875. Amuta et al. (2023) [24] utilized deep neural networks and binary butterfly optimization algorithms for cardiovascular disease prediction. Research made breakthroughs in accuracy and efficiency in predicting cardiovascular health using DL technology. In summary, this work adopted advanced neural network architecture and DL models to achieve accurate analysis and prediction of motion data through multi-level feature extraction and learning. Compared with previous ML models and related research, this work introduced neurorobotics technology into the aerobics intelligent fitness system, expanding the application prospects in intelligent fitness. By integrating big data and ML algorithms to deliver personalized fitness guidance to users, this work aimed to assist individuals in enhancing their quality of life, elevating their health status, and facilitating customized aerobic fitness training. At the same time, the relevant results in the previous research also provided a valuable reference for this work, offering the necessary support for developing the intelligent fitness field.

In the method section, this work explicitly states that all results and findings are original and have not been submitted or published elsewhere. This work emphasizes the uniqueness of the research, that is, the developed aerobics intelligent fitness system is based on neurorobotics technology, which is a relatively novel field. The purpose is to use big data and AI technology to design the aerobics intelligent fitness system of neurorobotics, use the fitness system to conduct physical fitness tests, and enhance the public's health awareness. Neurorobotics technology allows people to simulate the perception and control functions of the human brain, thus making fitness more intelligent. Firstly, neurorobotics technology is briefly described, which provides functions such as neural perception and control for the aerobics intelligent fitness system. Secondly, it discusses the relationship among big data, ML, big data technology products, and processes of ML, and focuses on calculating node data constructed by the Spark big data platform. Moreover, the decision tree algorithm is used for data preprocessing to provide data value for subsequent intelligent fitness analysis. Furthermore, the data performance and training effect of the system are analyzed. The research results of this work are independent and have not been submitted or published elsewhere, which can employ neurorobotics information data processing to fill further the gaps in neurorobotics and sports and fitness fields.

## 2 Method

### 2.1 Analysis of neurorobotics

**Fig. 1:**
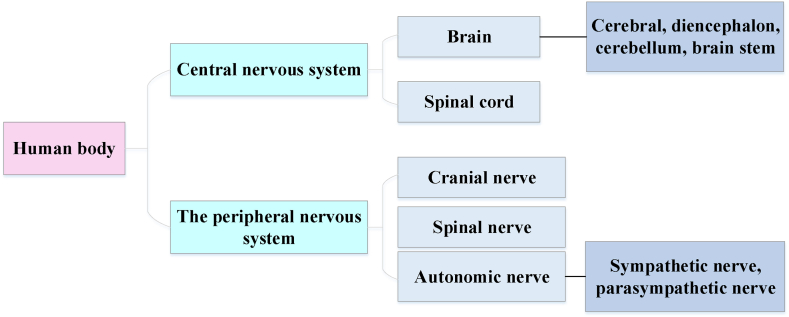
Components of the nervous system.

Neurorobotics, a core technology in this work, finds application primarily in intelligent fitness systems, where it simulates the perception and control mechanisms of the human brain to enhance system intelligence. Although the data collection process in this work does not directly involve experimental operations in neurorobotics, the principles and techniques of neurorobotics provide the theoretical foundation and algorithmic support for the system. Specifically, this work utilizes the Cixi 3.0 intelligent fitness facility (http://zjpubservice.zjzwfw.gov.cn/jyxxgk/002007/002007002/20210507/473ca543-bb91-489e-839b-e0672626a9d3.html) as a technical reference, which integrates intelligent interaction, facial recognition, and scientific fitness guidance—manifestations of neurorobotic technology in practical applications. Through an analysis of the design concept and functional implementation of Cixi 3.0, this work extracts key technological elements relevant to intelligent fitness systems and integrats them into the research model. Nervous systems encompass brain-inspired algorithms (such as connectionist networks), computational models of biological neural networks (such as artificial spiked neural networks and large-scale simulations of neural microcircuits), and actual biological systems (such as in vivo and in vitro neural networks). Such nervous systems can be embodied in machines with mechanical or other forms of physical actuation [25]. The composition of the nervous system is portrayed in Fig. 1.

**Fig. 2:**
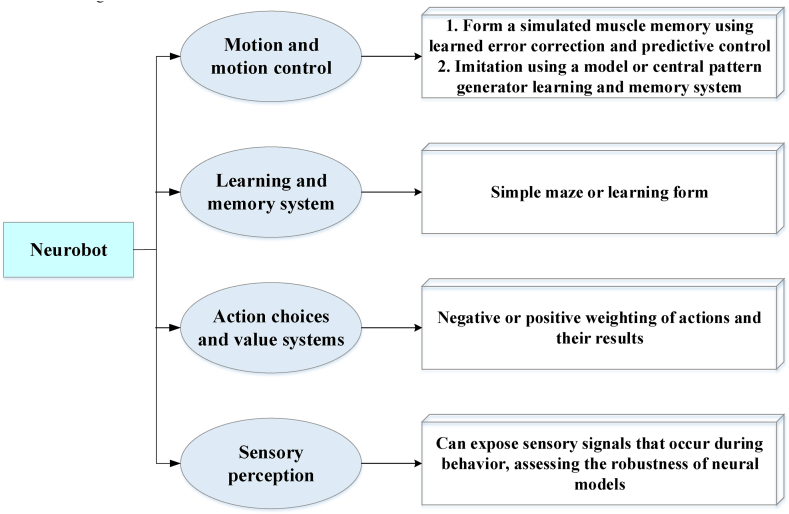
Categories of neurorobotics.

The central nervous system directs the movement of the limbs, trunk, and head. The command center of the peripheral nervous system is located in the center of the brain, which is connected to the delicate fibrous network on each side of the spine. Extensive nerve branches extend from this central hub, reaching various internal organs to regulate their functions. Although the body and brain design (“control”) are key factors in making robots move, existing benchmark environments only support the latter [26]. The evolution of robots has transitioned from industrial applications to integration into everyday life. Robots have shifted from stationary robotic arms performing repetitive tasks to dynamic, cooperative machines. This transition marks the development of robot “intelligence.” Cixi 3.0 smart fitness facility is intelligent fitness equipment with intelligent interaction, face recognition, and scientific fitness fun lights. It also has an intelligent fitness robot that can indicate the weather, measure blood oxygen and heart rate, and provide fitness guidance. Cixi 3.0 smart fitness facilities plan the open space reasonably, design fitness pathways, and provide citizens with personalized services, thus enabling them to experience the unique allure of “Internet + fitness.” A mechanical exoskeleton is a robot, a new technology for humans. People who are disadvantaged in walking hand over their bodies to machines. The robot moves forward at a constant speed. The movements and steps of each step are exactly the same [27]. The working systems of robots are founded on the principles of the nervous system. This includes robots, prosthetic or wearable systems, micro-machines on a small scale, and furniture and infrastructure on a large scale [28]. The core idea of neurorobotics is to embody the brain and embed the body into the environment. Therefore, most neurorobotics need to function in the real world instead of in simulated environments. In addition to robot-inspired algorithms, neurorobotics may also involve designing brain-controlled robotic systems. According to the robot's purpose, neurorobotics can be divided into those used to study motor control, memory, choice of action, and perception [29]. Fig. 2 demonstrates categories of neurorobotics.

In the specific implementation process, the integration of technology begins by merging neurorobotics technology with big data and ML technology to construct an intelligent fitness system relying on DL models. This system is designed to generate intelligent fitness recommendations and devise personalized training plans for users. Subsequently, users' exercise data, including key metrics such as steps, exercise time, and heart rate, are collected through smart fitness devices and apps. These data are then transmitted to the system in real-time for in-depth analysis. Ultimately, by combining the control features of neurorobotics with the user's specific physical condition and personal goals, a personalized fitness program is tailored to ensure that each user received the most suitable fitness guidance and advice. Through this series of steps, neurorobotics is successfully applied to a smart fitness system, enhancing not only fitness efficiency but also the overall user fitness experience.

### 2.2 Analysis of big data and ML technology

In general, big data technology and ML are mutually reinforcing and interdependent. ML requires reasonable, applicable, and advanced algorithms and high-quality data. Big data can improve the accuracy of ML models. The greater the amount of data, the higher the quality and the higher the accuracy and performance of ML. The core of big data is to utilize the value of data, and ML is to use the key technology of the data value. For big data, ML is indispensable. On the contrary, for ML, more data is more likely to improve the model's accuracy. Furthermore, the computing time of complex ML algorithms also urgently requires distributed computing and in-memory computing.

Big data is the collection of data that cannot be captured, managed, and processed by conventional software tools within a specific time frame. It is a massive, high-growth, diversified information asset requiring new processing modes with strong decision-making, insight discovery, and process optimization capabilities. The architecture of the big data technology product is displayed in Table 1.

**Table 1:** The architecture of the big data technology product.

Big data technology product	Underlying architecture	Application
Hadoop big data platform	Open source, reliable, scalable distributed system	Big data storage and computing
Spark big data platform	Memory-based distributed computing framework	Big data computing

Table 1 implies that the underlying architectures of Hadoop and Spark are different. Hadoop is an open-source framework to store and process large data in a distributed environment. It consists of two modules. One is MapReduce, and the other is Hadoop Distributed System (HDFS). MapReduce is a parallel programming model that can process largely structured, semi-structured, and unstructured data on large clusters of common hardware. HDFS is part of Hadoop's framework for storing and processing datasets. It provides a fault-tolerant file system running on common hardware. The Hadoop ecosystem contains different sub-project (tool) modules for assisting Hadoop, such as Sqoop, Pig, and Hive. Sqoop imports and exports data back and forth between HDFS and Relational Database Management System. Pig is a platform for scripting programming languages for developing MapReduce operations. Hive is a platform for developing Structured Query Language (SQL) scripting for MapReduce operations. Moreover, MapReduce jobs can be executed using various methods. The traditional approach uses Java MapReduce programs for structured, semi-structured, and unstructured data. For the scripted way of MapReduce, Pig is used to process structured and semi-structured data. Hibernate Query Language is to use Hive as MapReduce to process structured data [30].

Resilient Distributed Dataset (RDD) is the entire Spark's core and architectural foundation. It is a fault-tolerant and parallel data structure that allows users to explicitly store data on disk and memory and control data partitioning. RDDs also provide a rich set of operations to manipulate this data. Among these operations, transformation operations such as Map, flatMap, and Filter implement a functional programming pattern (monad pattern) that fits well with Scala's collection operations. In addition, RDD also provides convenient operations such as Join, GroupBy, and reduceByKey to support common data operations. Generally speaking, several common data processing models exist, including iterative algorithms, relational queries, MapReduce, and stream processing. For example, Hadoop and MapReduce adopt the MapReduce model, while Storm adopts the stream processing model [31]. RDD mixes these four models, and Spark can be applied to various big data processing scenarios. A comparison of Spark and Hadoop is exhibited in Table 2.

**Table 2:** Comparison of spark and hadoop.

Point of comparison	Spark	Hadoop
Ease of use	Standard SQL, easy to use	Support basic SQL
Performance	In-memory computing, 10–100 times faster than Hadoop	Slow
Graph computation	Support	Not support
ML	Mlib module	Not support
Community and activity	Index rises	Average
Application scale	The nodes are all in use, and the maximum cluster is 8000 nodes.	Less than spark

**Fig. 3:**
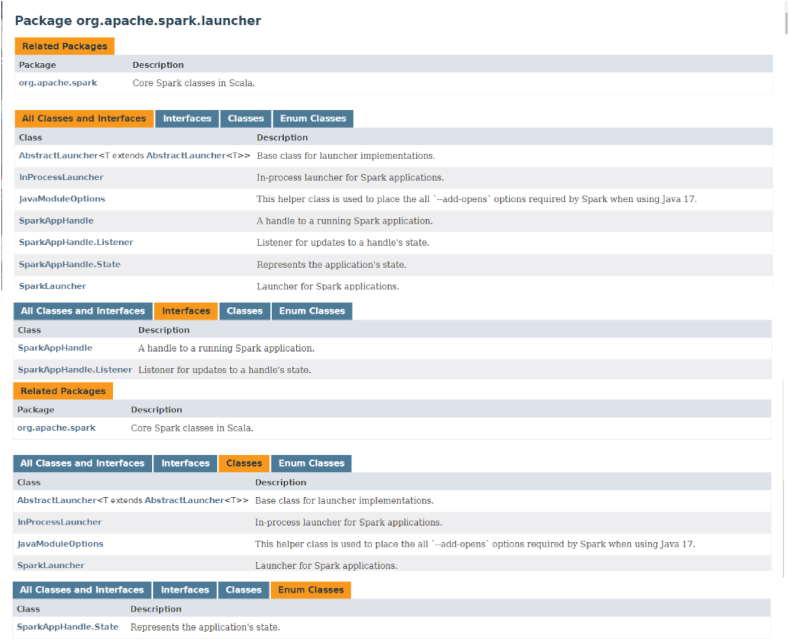
Information about RDD

By adopting the Spark-based RDD model as the main architecture, this work can fully utilize RDD's fault tolerance and parallelism advantages to effectively process and analyze large-scale data. In this experiment, a large amount of data is used from multiple data sources, including motion data collected by sensors, fitness records provided by users, and the use of public fitness facilities. These data are considered big data because of their large scale and complex structure. However, it should be noted that the dataset of this work is based on a limited number of people, so there are certain limitations in studying the requirements behind big data systems. Future research can further explore and apply a broader range of data sources to better understand and meet big data processing needs. The relevant information of the RDD dataset used in this work is plotted in Fig. 3.

**Fig. 4:**
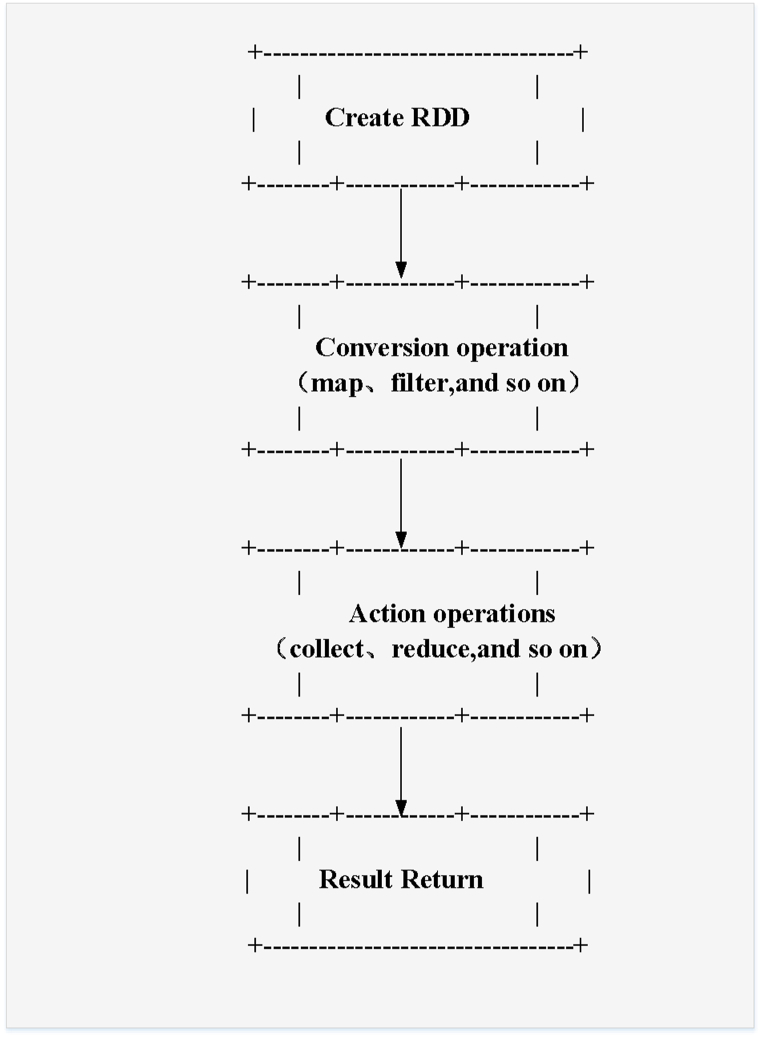
RDD process.

The Mlib module in Table 2 functions similarly to ML libraries such as Scikit-Learn, but the computing engine uses Spark. All computing processes are distributed. ML is a branch of computer science that allows computers to learn without being explicitly programmed. The RDD process is illustrated in Fig. 4.

Fig. 4 illustrates the significance of RDDs as distributed data sets and demonstrates how data can be processed and manipulated through transformations and actions. RDDs can be generated from external data sources like files, databases, etc., or derived from existing RDDs by applying transformations. Transformations are applied to each element in the RDD, resulting in a new RDD. These operations, such as map and filter, are utilized for data processing and filtering. Actions trigger computations on RDDs and yield results. Examples of actions include collect and reduce, which are used for aggregating data or returning specific results. The computed results are then either returned to the user or stored in external data sources.

The essence of building an aerobics intelligent fitness system using neurorobotics technology, big data analytics, and ML lies in harnessing advanced technologies to deliver personalized fitness experiences to users. Neurorobotics technology furnishes the system with neural perception and control capabilities essential for understanding users' movement status and preferences, enabling the design of personalized fitness plans. Meanwhile, big data analytics and ML empower the system to process and analyze user data, identifying fitness patterns, habits, and preferences to offer tailored fitness recommendations. By integrating these technologies, an intelligent fitness system can be developed to better cater to users' fitness needs, aiding in lifestyle improvement and health enhancement. In employing the Spark big data platform and decision tree algorithm for data preprocessing and intelligent fitness analysis, the Spark platform constructs node data for computation, while the decision tree algorithm preprocesses the data. The decision tree algorithm, an intuitive ML method, categorizes datasets into different branches based on features, facilitating data preprocessing. Leveraging Spark's RDD model as the primary architecture enables effective processing and analysis of large-scale data, capitalizing on RDDs' fault tolerance and parallelism advantages. The system architecture encompasses coach algorithm matching and functional value calculation implementation. Through ML algorithms, the system matches users with suitable coaches based on their physical condition and goals. It also evaluates users' movement posture and performance comprehensively, providing professional fitness advice using big data analytics and ML. Additionally, personalized fitness plans based on individual circumstances enhance workout effectiveness and efficiency. By employing these design and implementation methods, the aerobics intelligent fitness system delivers personalized fitness plans and guidance, making the fitness process more intelligent, convenient, and user-centric. It records real-time exercise status, receives workout reports, and builds user-centered intelligent fitness systems, promoting more effective aerobic fitness training. For smaller-scale datasets, simpler data processing techniques like Pandas are utilized to enhance analysis efficiency and streamline implementation processes.

**Fig. 5:**
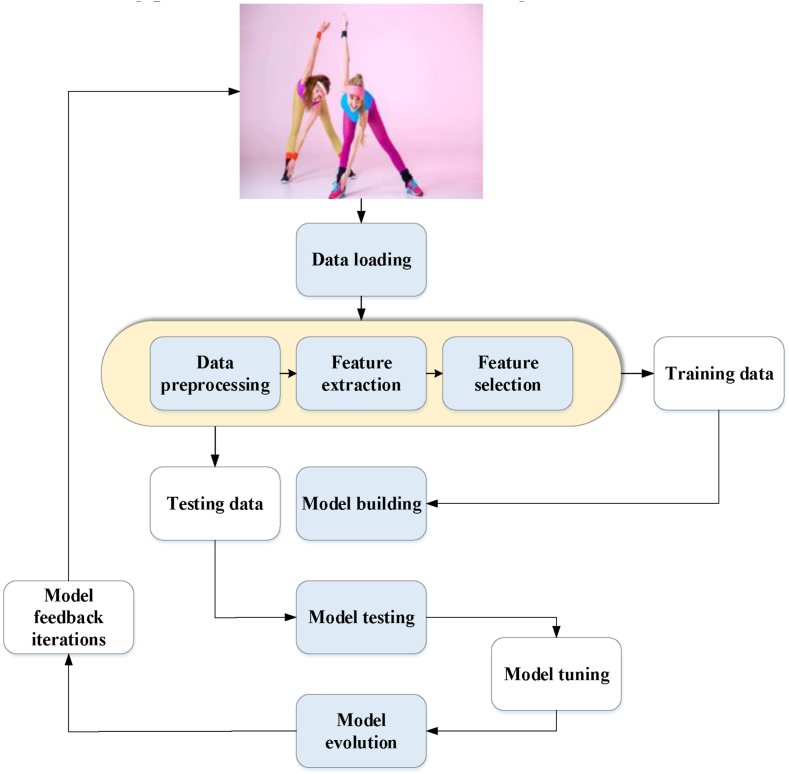
The working process of ML.

ML explores the study and construction of algorithms for heuristic learning and making predictions based on data. This algorithm implements models from sample inputs, replacing strictly static program code by formulating data-driven predictions. Typical ML tasks are concept learning, predictive modeling, clustering, and finding useful patterns. The ultimate goal is to increase learning automation so that human intervention is no longer required, or the level of human intervention is reduced as much as possible [32]. The working process of ML is revealed in Fig. 5.

The process of ML starts by loading sample data and parsing the data into the format required by the algorithm. The test dataset is randomly split and is usually divided into a training dataset and a test dataset called sampling. The training and test datasets are used to train the model and evaluate the best model's performance [33].

In this work, the amalgamation of big data techniques and ML greatly augments the performance of the smart fitness system. The following technical applications and approaches are adopted to achieve this objective: Firstly, a dependable distributed system is constructed using the Hadoop and Spark big data processing platforms as the cornerstone of the data architecture. This system adeptly stores and computes large volumes of data, laying a robust groundwork for subsequent analysis and model training. Secondly, during the data processing stage, Spark's in-memory computing framework is leveraged. The efficiency of this framework facilitates the rapid processing and analysis of the extensive motion data collected, enabling the extraction of valuable insights and patterns. Lastly, for model training and optimization, Spark's MLlib module for distributed machine learning training is employed. This module not only enhances model training efficiency but also substantially boosts the prediction accuracy of the system by optimizing model parameters. Through these technical applications and methods, the high performance and reliability of the intelligent fitness system in processing and analyzing big data are ensured, thus providing users with more precise and personalized fitness services.

### 2.3 Design of aerobics intelligent fitness system

**Fig. 6:**
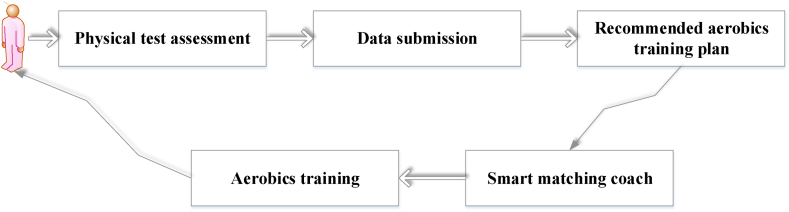
Design of aerobics intelligent fitness system.

At present, smart sports and fitness have covered both online and offline product forms. Online products take the mobile application as the main form, supplemented by a wealth of fitness courses, community exchanges, live broadcast interaction, and other content. Smart sports and fitness apps can provide users a relatively complete digital smart fitness experience. In terms of courses, the body data entered by users can recommend personalized and effective courses, create meal plans, and improve fitness efficiency. Regarding coaching resources, the professional coaches contracted by the app are cost-effective, which can avoid the problem of uneven personal training in offline gyms. Aerobics smart fitness system is designed through neurorobotics, big data, and ML. Before using the system, users need to input their fitness data and retrieve it through the system sensors to generate the optimal smart fitness plan. Fig. 6 indicates the design of a smart fitness system for aerobic exercise.

**Fig. 7:**
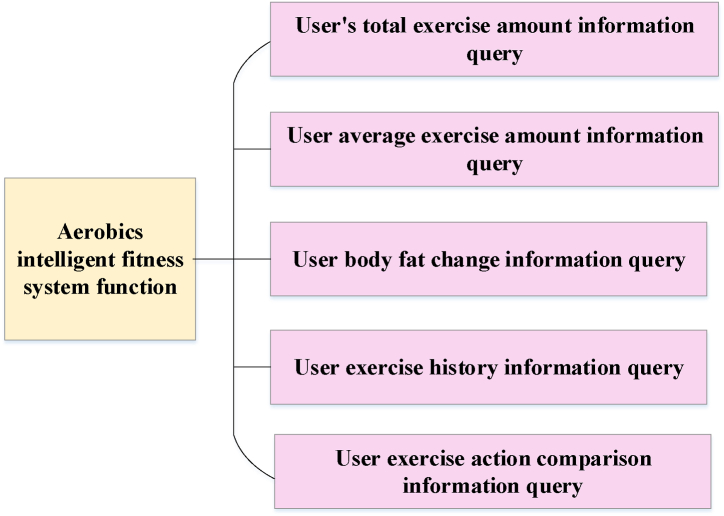
Realization function of aerobics intelligent fitness system.

In the intelligent fitness system shown in Fig. 6, algorithms are utilized to calculate the matching of coaches and the values of implementation functions. The system harnesses ML algorithms to facilitate coach-user matching, which hinges on the user's physical profile, health condition, and individual objectives. The system can analyze big datasets and find the most suitable coach for user needs by comparing user characteristics and factors such as the coach's professional domain and experience. To calculate the value of the implementation function, the system comprehensively evaluates the user's exercise posture and performance based on the fitness data entered by the user and the data collected by the sensor, combined with big data analysis and ML algorithms. Moreover, the system analyzes these data and applies advanced algorithms and scientific exercise suggestions to provide professional exercise advice for aerobic fitness users. These suggestions will be personalized based on the user's situation to improve exercise effectiveness and fitness efficiency. Through the above calculation explanations and personalized functions, the intelligent fitness system can offer customized exercise plans and guidance according to user needs and conditions, facilitating more effective engagement in aerobic fitness training. The functions of the aerobics intelligent fitness system are illustrated in Fig. 7.

The aerobics intelligent fitness system can enable users to enjoy personalized customized scenarios during the fitness process, record real-time exercise status, receive exercise reports, and create a fitness intelligent system centered on user needs, making fitness exercise intelligent, convenient, and user-centric.

The decision tree model is employed to preprocess the data of aerobic fitness users, mine the data value, and provide data value for the subsequent intelligent fitness analysis [34]. The decision tree uses the idea of classification to implement a mathematical model according to the characteristics of the data, and the computer judges the right and wrong of the result. The main feature of the decision tree model is its intuitive and easy-to-understand structure. The model consists of a series of decision and leaf nodes, each decision node representing a feature or attribute, and the dataset is classified into different branches based on these features. A tree structure is obtained by gradually dividing each branch, allowing samples to be correctly classified into corresponding leaf nodes. Moreover, the decision tree model also has the advantage of strong interpretability, and the generated rules can be interpreted as feature weights and association rules, which contributes to understanding and inferring the data's potential patterns and feature importance.

(1) $$H_{0}\left(x\right)=G\left(0^{T}x\right)$$

(2) $$G_{0}\left(z\right)=\frac{1}{1+e^{-x}}$$

In Eq. (1)- Eq. (2), x and z express eigenvectors, which are used to represent the results. G(z) refers to the correlation between the output function value and the threshold value in an interval. The value of z can be calculated by transposing, and the result can be obtained [35]. When G(z)=0.5, z=0. Min-Max normalization is adopted to improve the accuracy and speed of model calculation.

(3) $$y=\frac{x-\min}{\max-\min}$$

In Eq. (3), the feature sequence $x_{1},x_{2},\cdots ,x_{n}$ is transformed. y stands for the transformed, new sequence, and y∈[0,1]. min denotes the minimum value of the input sample $x_{i}$, and max signifies the maximum value of the input sample $x_{i}$. While entering aerobic fitness data, the machine model can re-normalize the data [36]. Z-Score standardization refers to the standardization of data by the mean and standard deviation of the original data.

(4) $$y=\frac{x-\mu }{\sigma }$$

In Eq. (4), μ represents the mean of the total sample, and σ indicates the standard deviation. Data of different magnitudes can be unified into the same magnitude, and the calculated Z-Score value can be used to measure the data, which ensures comparability between the data. The information gain is used to determine the effect of the nodes built in the big data technology environment.

(5) $$\text{Gain}\left(S,A\right)=\text{Entropy}\left(S\right)‐\sum_{v\in Values\left(A\right)}^{A}(\frac{S_{v}}{\left|S\right|}*\text{Entropy}\left(S\right)$$

(6) $$\text{Entropy}\left(S\right)=‐\sum_{i=1}^{n}p_{i}\;\mathrm{Log}\left(p_{i}\right)$$

In Eq. (5) - Eq. (6), Entropy(S) demonstrates the information entropy, which is similar to the probability summation of *n* independent repeated experiments. The smaller the Entropy(S), the higher the collection purity of the samples selected here. S shows the record value of all root nodes. v displays the attribute value of the selected sample data. $S_{v}$ means the information entropy of each attribute. Information gain results in a large number of sample attribute values. This problem can be written as Eq. (7).

(7) $$\text{GainRatio}\left(S,A\right)=\frac{Gain\left(S,A\right)}{IntrinsicValue\left(A\right)}$$

In this work, an aerobics smart fitness system was meticulously designed to achieve seamless integration and application of technology through a series of carefully crafted steps. Initially, users are prompted to input their personal fitness data, while the system simultaneously collects real-time data through sensors. This amalgamated dataset is then utilized to generate an optimal personalized smart fitness plan tailored to each user's requirements. Subsequently, ML algorithms are employed to intelligently pair trainers with users and compute the specific value of implemented features, ensuring that individualized fitness guidance is provided to meet users' unique needs. Moreover, the system is endowed with real-time monitoring capabilities, enabling continuous tracking and recording of users' exercise status. Timely exercise reports are generated, culminating in the development of a user-centric intelligent fitness system. Through these meticulously executed steps, the effective integration and application of technology are assured, ultimately offering users an efficient, personalized, and interactive fitness experience.

ML classification models are evaluated by calculating the precision, recall, precision, and F1 (H-mean) values [37].

(8) $$P\left(precision\right)=\frac{TP}{TP+FP}$$

(9) $$R\left(recall\right)=\frac{TP}{TP+FN}$$

(10) $$A\left(accuracy\right)=\frac{TP+TN}{TP+TN+FP+FN}$$

(11) $$F1=\frac{2TP}{2TP+FP+FN}$$

In Eq. (8)- Eq. (11), TP and TN stand for the number of actual positive and negative classes predicted to be positive and negative. FP and FN manifest the number of actual negative and positive classes predicted to be positive and negative. Eq. (8) indicates the probability of being a positive sample in the predicted positive sample. Eq. (9) represents the probability that the actual positive sample is predicted to be a positive sample, and it implies the probability that a positive sample is all the samples in the predictions. The smaller the value of F1 in Eq. (11), the smaller the sample error rate [38]. The algorithm flow code of the aerobics intelligent fitness system is denoted in Table 3. Analyzing the computational cost of the algorithm involves several factors. Firstly, it computes the current fitness value for each particle, necessitating the evaluation of each particle's position and the fitness function. Secondly, it calculates gradients by performing gradient descents across the entire dataset. Additionally, it computes the value of the Salomon function by determining the position of each particle. Furthermore, operations such as initializing historical and current best positions and updating the time step entail data computation and processing, contributing to the algorithm's high computational cost. For instance, if the algorithm utilizes 100 particles, each requiring one fitness function evaluation, a total of 100 evaluations are necessary. Similarly, computing gradients entails calculations across the entire dataset. If the dataset contains 1000 samples, each gradient descent operation involves computation across all 1000 samples, resulting in 100 computations. Likewise, computing the Salomon function requires determining the position of each particle, totaling 100 computations. Additionally, operations like initializing historical and current best positions and updating the time step involve corresponding computations. Therefore, the algorithm incurs a high computational cost, particularly evident with large-scale datasets, where the computational overhead becomes more pronounced.

**Table 3:** The algorithm flow code of the aerobics intelligent fitness system.

1	**Algorithm:** Aerobics Intelligent Fitness System
2	**Require:** User's fitness data
3	**Require:** Step length ε (default 0.001)
4	**Require:** Exponential decay rate of moment estimation ρ1, ρ2
5	**Require:** Constant of numerical stability α
6	**Require:** Initial parameter θ
7	**Initialize** the historical optimal position Pi and current optimal position Pg
8	Initialize time t = 0
9	**While** Stop criteria not reached do
10	Calculate the current fitness of all particles
11	Compute gradient g: g ← 1/m ∇θ∑_i L (f (xi; θ), yi)
12	Calculate function value Salomon:
13	Salomon ← 1 - cos (2π*(∑_(i = 1)^n xi^2)) + 0.1*(∑_(i = 1)^n xi^2)
14	Partial first-order moment estimation: s ← ρ1s + (1-ρ1)g
15	Partial second-order moment estimation: r ← ρ2r + (1-ρ2)g⊙g
16	Correction of first-order moment deviation: s_tilde ← s/(1-ρ1^t)
17	Correction of second-order moment deviation: r_tilde ← r/(1-ρ2^t)
18	Calculation update: Δθ ← -ε s_tilde/(√(r_tilde) + α)
19	Apply updates: θ ← θ + Δθ
20	Increment time: t ← t + 1
21	**End while**

### 2.4 Experimental data and environment

The research dataset in this work comprises motion data collected from sensors and fitness records provided by users, facilitated through smart fitness devices and fitness applications. Motion data, including step count, exercise time, and heart rate, is monitored in real-time by smart fitness devices and transmitted to the system for analysis of users’ exercise habits and health conditions. User-provided fitness records, obtained through distributed surveys via Amazon Mechanical Turk, detail minute-level outputs of physical activity, heart rate, and sleep monitoring. The data is sourced from https://www.kaggle.com/datasets/arashnic/fitbit. Data preprocessing involves several methods tailored to different data types and characteristics. For motion data, initial cleaning removes outliers and missing values, followed by standardization to unify data collected by diverse sensors to a consistent scale for analysis. Fitness records undergo format conversion and annotation to ensure consistency and availability. Data augmentation techniques, including synthesis and augmentation, are employed to enhance diversity and representativeness. In handling large-scale datasets, distributed computing and parallel processing techniques are utilized to optimize efficiency and processing speed.

The system utilizes Hadoop 3.0 for performance support and optimization, operating within a Linux environment. Hardware configuration includes a Master Node, featuring an Intel i3 dual-core processor and 16 GB of memory, coordinating the Spark cluster. Worker Nodes consist of Node 1, equipped with an Intel i7 hexa-core processor and 32 GB of memory, and Node 2, utilizing an AMD Athontm II dual-core processor and 16 GB of memory. These nodes collectively form the Spark cluster for distributed processing of large-scale data. Software environment comprises Spark version 3.0 for distributed computing and data processing, alongside the Hadoop Distributed File System (HDFS) for storing large-scale datasets. Network connectivity ensures fast and stable communication among nodes to facilitate data transmission and collaborative execution of distributed computing tasks. Monitoring and management tools are employed to monitor operational status, resource utilization of cluster nodes, and promptly identify and resolve potential issues. Additionally, these tools facilitate management of cluster configurations, node deployment, software installation, and updates.

## 3 Results analysis

### 3.1 Analysis of the evaluation results of the aerobics intelligent fitness system model

**Fig. 8:**
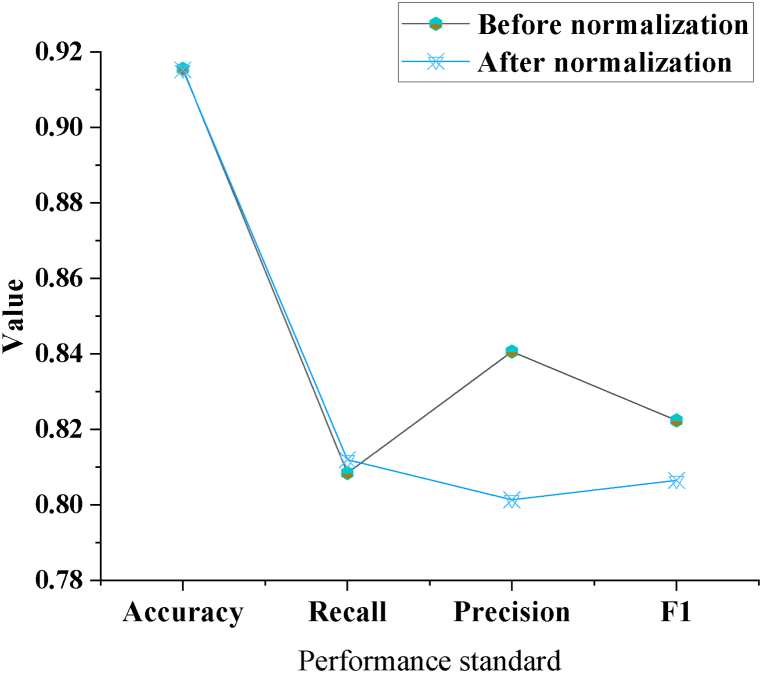
Analysis of the evaluation results of the aerobics intelligent fitness system model.

It uses the aerobic fitness data on the Android version of Smart Fitness v1.0.8 as the research model performance and 35 % of the aerobic fitness data is selected as the dataset. Fig. 8 expresses the analysis of the evaluation results of the aerobics intelligent fitness system model.

In Fig. 8, the proposed intelligent fitness system model achieves an accuracy of 91.52 %, with a recall rate of 81.19 %, precision of 80.13 %, and an F1 value of 80.64 %. Subsequently, this section conducts an analysis comparing the performance of data normalized using Min-Max normalization with unnormalized data. Results indicate a slight decrease in the performance of Min-Max normalized data compared to unnormalized data, as follows: Min-Max normalized data accuracy is 91.52 %, while unnormalized data accuracy is 91.55 %. The recall rate of Min-Max normalized data is 80.84 %, whereas for unnormalized data, it is 81.19 %. Min-Max normalized data precision is 84.06 %, in contrast to unnormalized data precision of 80.13 %. Additionally, the F1 value of Min-Max normalized data is 82.23 %, while for unnormalized data, it is 80.64 %. Analysis of the results indicates that Min-Max normalized data, compared to unnormalized data, showed slight improvements in accuracy and precision but slight decreases in recall rate and F1 value. This could be attributed to the Min-Max normalization method, which confines data within a range of 0–1, potentially leading to limitations in data variation range and consequent information loss.

Min-Max normalization is a widely used data preprocessing technique that scales raw data to a defined range, typically between 0 and 1, for standardization. Its primary aim is to mitigate the influence of disparate scales, ensuring each feature contributes proportionately to enhance ML model performance. Normalizing data aids in accelerating optimization algorithm convergence, such as gradient descent, as all features share a common scale, preventing instances where certain features disproportionately affect the loss function due to large numerical values. Variations in input variable numerical values can destabilize or complicate model training, a challenge alleviated by Min-Max normalization. However, since Min-Max normalization adjusts data based on minimum and maximum values, its efficacy may diminish in the presence of outliers. Outliers extend the data scale, compressing most values into a narrow range. In some instances, the original data distribution, such as non-uniformity, holds significance for the model, which normalization could obscure. For instance, in this work of the intelligent fitness system, employing both Min-Max normalized and unnormalized data for model training and testing reveals a decline in the F1 value of normalized data. While normalization aids in enhancing model generalization, it may reduce sensitivity to data, thereby impacting accuracy. Furthermore, normalized data exhibits a lower error classification rate than unnormalized data, evident through comparative analysis of accuracy, recall rate, and F1 value under both processing methods. Min-Max normalization mitigates overfitting risks from extreme values by adjusting data scale, enabling better capture of overall data distribution characteristics. However, its suitability depends on the dataset's characteristics. For datasets with significant outliers, given this normalization method's high sensitivity to extreme values, preprocessing outliers or opting for less sensitive techniques, such as z-score standardization, may be necessary. Consequently, while Min-Max normalization enhances model training efficiency and generalization, its sensitivity to outliers poses limitations. Hence, in practical applications, flexible selection of data preprocessing methods based on specific data traits and model requisites is crucial. This analysis underscores normalization's positive impact on intelligent fitness system performance, particularly concerning maximum or minimum value influences on model outcomes, while urging careful consideration of data preprocessing steps' selection and application.

**Fig. 9:**
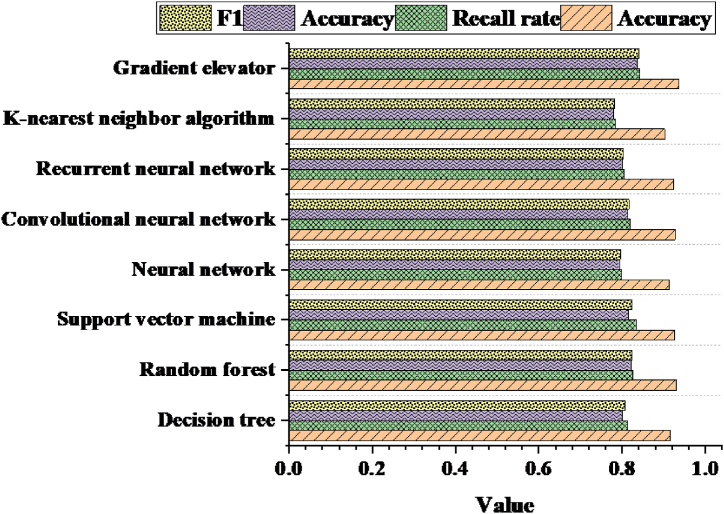
Performance comparison of each algorithm across accuracy, recall, precision, and F1 value metrics.

To assess the performance of the proposed decision tree algorithm in the aerobics intelligent fitness system, this work compares it with several commonly employed ML algorithms, including Random Forest, Support Vector Machine (SVM), Neural Network, CNN, Recurrent Neural Network (RNN), K-Nearest Neighbors (KNN), and Gradient Boosting Machine (GBM). The performance of each algorithm is evaluated by employing the same dataset for both training and testing. Fig. 9 illustrates the performance of each algorithm in terms of accuracy, recall rate, precision, and F1 value. From Fig. 9, it is evident that while the proposed decision tree algorithm demonstrates commendable performance across accuracy, recall rate, precision, and F1 value metrics, it does not outperform all others in every aspect. Random Forest and GBM particularly excel across all metrics, particularly in accuracy and F1 value. In contrast, the decision tree algorithm exhibits slightly inferior performance in terms of recall rate and precision.

### 3.2 Analysis of the physique test effect of aerobics intelligent fitness system

**Fig. 10:**
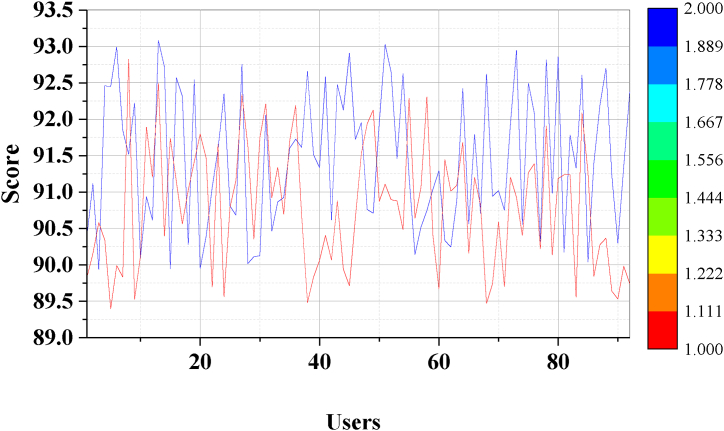
Analysis of the physique test effect of aerobics intelligent fitness system.

The aerobics intelligent fitness system is designed through neurorobotics, big data, and ML. To ensure optimal learning from a comprehensive dataset and bolster the model's generalization to unseen data, this work intends to utilize the complete dataset comprising data from all 92 users for both model training and testing. This approach mitigates the risk of bias or inaccuracies stemming from random data subset selection. The test content is the user's body fat, body shape changes, vital capacity, flexibility, limb coordination, changes in endocrine regulation function, and the acuity of the human brain, vision, and hearing under the nervous system during the intelligent fitness process of aerobics. Given the intricate nature of quantifying each factor, this work adopts a holistic approach to assessing these variables, systematically predicting and scoring the users' physical fitness. The physical fitness test effect analysis of the aerobics intelligent fitness system is revealed in Fig. 10.

During the evaluation and validation process of this work, Fig. 10 illustrates that prior to system testing, the users' average actual comprehensive physical fitness score stands at 91.40 points. Post-system testing, the average predicted score is 90.91 points. By analyzing the disparities between the system's predictions and actual training outcomes, minimal differences are observed, ranging approximately from 3.13 points. This suggests that the intelligent fitness system's training has no significant impact on overall physical fitness, aligning with users' expectations.

In summary, this work presents the design of an intelligent fitness system leveraging ML and distributed system architecture, yielding satisfactory evaluation results. The findings suggest that integrating ML and distributed system architecture into fitness system design enhances model evaluation accuracy and optimizes system design. While the sample size in this work is relatively small and may not fully represent typical big data characteristics, the intelligent fitness system's architecture is built on technology capable of handling and analyzing large-scale datasets beyond the 92-user sample. The system design accounts for processing requirements of larger volumes, rapidly changing, and diverse data types anticipated in the future. The aim of this architecture and algorithm design is to ensure effective data processing and analysis in future big data environments, thereby delivering more accurate and personalized intelligent fitness services. Although the evaluation in this work utilizes a small dataset, the system's operation and performance are not hindered by larger-scale datasets. The system architecture and algorithm design exhibit scalability and adaptability, facilitating seamless handling of larger-scale datasets. For instance, multi-source data, including sensor motion data, user fitness records, and public fitness facility usage patterns, are collected and extensively utilized for system training and optimization. Moreover, the system supports large-scale parallel computing and real-time data processing, enabling efficient handling of massive data volumes and rapid responses. Leveraging distributed computing and parallel processing technologies ensures high efficiency and stability when dealing with large-scale data. Therefore, despite the limited sample size, confidence exists that in future big data environments, the intelligent fitness system will adeptly handle larger and more complex data processing tasks, offering users an enhanced fitness experience.

**Fig. 11:**
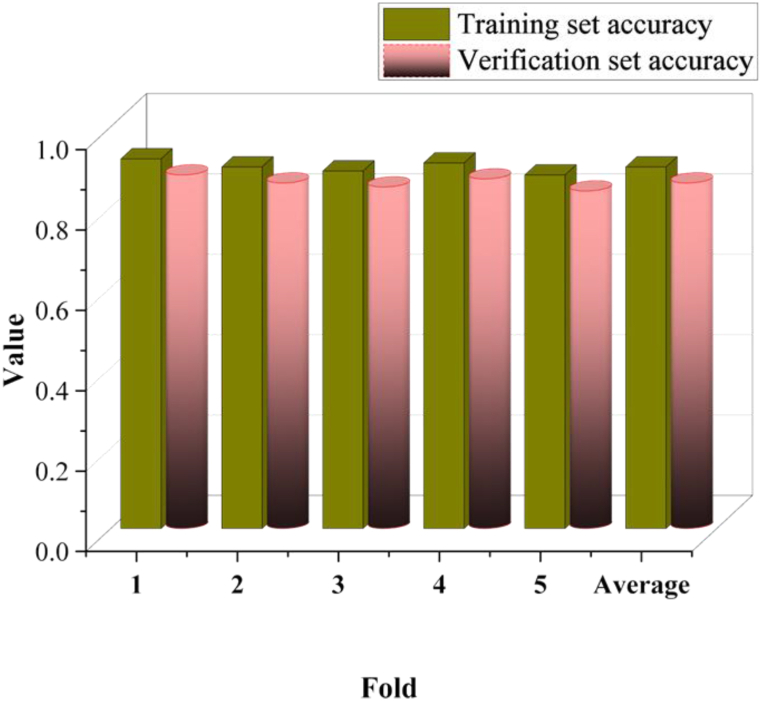
Model performance evaluation results using cross-validation technique.

To address potential computational resource constraints and conduct a robust evaluation of the model's performance, this work implements cross-validation techniques. Cross-validation involves using different subsets of the data to assess the model's performance without discarding any observations. This method ensures the model's exposure to the entire dataset while systematically and comprehensively evaluating its performance. Specifically, k-fold cross-validation is employed, dividing the dataset into k subsets. One subset serves as the validation set, while the remaining k-1 subsets trained the model. This process iterated k times, with each subset taking turns as the validation set. The average of the k validation results provided the model's final performance evaluation metric. Fig. 11 presents the results of evaluating the model's performance using a k-value of 5 in the cross-validation technique.

The cross-validation results depicted in Fig. 11 reveal the model's accuracy on both the training and validation sets. The average accuracy on the validation set, at 0.86, suggests the model's strong generalization ability and its capability to make accurate predictions on unseen data. Moreover, the minimal disparity in accuracy between the training and validation sets indicates the model's resilience against overfitting or underfitting concerns. Thus, utilizing cross-validation techniques for performance evaluation offers a thorough and precise assessment of the model's capabilities, affirming its robust generalization ability.

### 3.3 User survey and physical health examination results of fitness system

This work conducts a survey among users of the fitness system to evaluate their experience and any changes in their health status. The survey includes actual users of the system, ensuring diversity in age, gender, and health conditions to enhance the credibility of the findings. A random sampling approach is utilized to select 200 participants from the user pool, ensuring the sample's representativeness. The users provide feedback on their experience, satisfaction with the system's functionalities, and the user interface's friendliness, as summarized in Table 4.

**Table 4:** Feedback on usage experience, functionality satisfaction, and interface friendliness of the fitness system.

Item	Average/Frequency/Count/Proportion
Usage Experience Rating (1–10)	Average: 8.5
Functionality Satisfaction Rating (1–10)	Average: 9.0
Interface Friendliness Satisfaction	Yes: 180 (90 %)
	No: 20 (10 %)

The results of monitoring and analyzing users’ sustained engagement with the fitness system over time are presented in Table 5.

**Table 5:** Tracking and analysis of users’ long-term adherence to the fitness system during usage.

Item	Average/Frequency/Count/Proportion
Duration of Fitness System Usage (months)	Average: 9.0
Adherence to Usage	Yes: 190 (95 %)
	No: 10 (5 %)

The alterations in health metrics before and after users’ engagement with the fitness system are outlined in Table 6.

**Table 6:** Changes in health indicators before and after users’ use of the fitness system.

Item	Average/Frequency/Count/Proportion
Exercise Frequency (times/week)	Average: 4.5
Weight Change	Decrease: 160 (80 %)
	No Change: 30 (15 %)
	Increase: 10 (5 %)
Systolic Blood Pressure (mmHg)	Average: 120
Diastolic Blood Pressure (mmHg)	Average: 80
Heart Rate (beats/minute)	Average: 70

After comprehensive analysis of the data from Table 4, Table 5, Table 6, the following conclusions can be drawn: The average user experience rating and functionality satisfaction rating stand at 8.5 and 9.0, respectively, reflecting high user satisfaction with the fitness system's usability and features. A notable 90 % of users express satisfaction with the interface's friendliness, indicating positive reception of the interface design. A remarkable 95 % of users exhibit sustained long-term engagement with the fitness system, with an average usage duration exceeding 9 months, highlighting the system's strong appeal and sustainability. Eighty percent of users report weight loss, while systolic and diastolic blood pressure levels remain within normal ranges, and heart rate stays appropriate, suggesting positive impacts on users' physical health. In summary, users exhibit high satisfaction and sustained usage of the fitness system. Moreover, there is evidence of improved physical health among users, underscoring the system's effectiveness in enhancing user experience and promoting health benefits.

### 3.4 Results and discussion

To sum up, based on big data and ML technology, an aerobics intelligent fitness system is designed and operated by a computer with a Linux system. The results of the system simulation on the Android version of intelligent fitness v1.0.8 display that the evaluation accuracy of the system model is 91.52 %. The recall, accuracy, and the F1 value are 81.19 %, 80.13 %, and 80.64 %. The minimum difference between the predicted value of the system and the actual score after performing aerobics intelligent fitness training through the system is about 1.30 points, and the difference between the maximum values is about zero points. Additionally, the aerobics intelligent fitness system proposed in this work, integrating neurorobotics technology and big data analytics, offers significant advantages over traditional fitness methods. Conventional approaches often lack personalized guidance and targeted optimization recommendations, limiting their ability to address diverse fitness needs. In contrast, the proposed intelligent fitness system leverages advanced technologies, amalgamating neural perception and control functionalities with big data and ML techniques to furnish users with tailored fitness plans and optimization suggestions, thereby better accommodating varying user needs and lifestyles. Compared to existing intelligent fitness systems like the Cixi 3.0, the proposed system boasts enhanced comprehensiveness and intelligence in functionality. Beyond basic features such as intelligent interaction, facial recognition, and scientific fitness guidance, this system utilizes decision tree algorithms for data preprocessing and harnesses the robust computing capabilities of the Spark big data platform for large-scale fitness data processing. This design facilitates more effective analysis and processing of user fitness data, culminating in more precise and personalized fitness recommendations, thus aiding users in achieving their fitness objectives. Furthermore, this work evaluates the efficacy of the system's user physical fitness testing, validating its practicality and effectiveness. In contrast to traditional methods, the proposed system offers comprehensive assessments of users' physical condition and health indicators, furnishing them with scientific and effective fitness solutions. Consequently, the aerobics intelligent fitness system proposed herein holds substantial advantages and promising application prospects in addressing obesity and suboptimal health issues prevalent in today's fast-paced, high-stress lifestyles. In the context of previous studies, Panicker et al. (2019) researched ML for physiological-based psychological stress detection systems to conduct a comprehensive investigation of the role of ML in emotion detection systems and stress detection systems, and the reliability and accuracy of the systems were verified [39]. Chen et al. (2019) developed and designed ML models for healthcare and showed that rapid advances in ML provide opportunities to improve clinical decision-making [40]. Mustafa et al. (2021) studied automated ML models in healthcare and clinical medical record analysis and investigated previous work on ML in healthcare. The results indicated that ML could reduce the burden on healthcare systems [41]. Therefore, using ML and distributed system architecture to design a fitness system can enhance the accuracy of model evaluation and further optimize the design of the fitness system.

Implementing big data and ML technologies, particularly for smaller organizations or projects, poses various challenges and difficulties. Firstly, establishing and maintaining large-scale data infrastructure entails substantial financial investment, covering costs associated with data storage, processing, and security. Such expenses can be burdensome for smaller entities that may lack the financial resources to afford them. Secondly, training and optimizing ML models demand significant computational resources, necessitating high-performance computers and a skilled team of professionals. Smaller organizations or projects may struggle to acquire and maintain these resources due to capacity or resource limitations. Moreover, expertise and skills in ML are in high demand, requiring proficiency in mathematics, statistics, and computer science. For non-specialized teams, acquiring these skills may pose a barrier. To address these challenges, smaller organizations or projects can explore external collaborations or resource sharing. Collaborating with larger enterprises, research institutions, or cloud computing service providers can facilitate sharing data infrastructure and computational resources, thereby reducing implementation costs and thresholds. Additionally, leveraging open-source big data and ML tools and platforms can be beneficial. These resources often offer pre-trained models and algorithms, expediting technology implementation while mitigating costs and risks. Furthermore, emerging technological trends like edge computing and automated ML offer more flexible and efficient solutions for smaller entities. Edge computing minimizes data transmission and storage costs by processing and analyzing data closer to the source. Meanwhile, automated ML streamlines the development and optimization of ML models, lessening reliance on specialized expertise.

## 4 Conclusion

This work utilized neurorobotics technology to offer neural perception and control functions for the aerobics intelligent fitness system. At the same time, by combining big data and ML technology, the decision tree and the Spark big data platform were used for data preprocessing and node data modeling. These methods and technologies provide an important foundation for future intelligent fitness analysis. Research results have found that data normalized using Min-Max has lower accuracy, recall, and F1 values, reducing misclassification. In addition, the intelligent fitness system has a significant improvement effect on the overall physical fitness of users. After system testing, the average predicted score is 90.88, with an average score of 91.44. After using the system, users’ overall physical fitness improvement effect is significant, which can meet their needs. However, there is no standardized measure of the data for different fitness levels.

While this work integrates advanced technologies such as neurorobotics, big data, and ML in designing the aerobics intelligent fitness system, there are still some limiting factors. Firstly, the system model used in this work relies on specific algorithms, such as decision tree algorithms, which may perform well in handling certain types of data or addressing specific problems but may have limitations in other scenarios and may not be entirely applicable to all situations. Therefore, future research could explore and introduce more diverse types of algorithms to enhance the system's applicability and performance. Furthermore, despite the limitations posed by the dataset's size in this work, the application of big data technologies remains pivotal for research endeavors. Firstly, the robust data processing capabilities afforded by big data technologies allow for efficient handling and analysis of large-scale datasets sourced from multiple channels. While the current dataset size may not be expansive, the architectural framework and algorithmic infrastructure employed are primed to accommodate future expansions in data volume and broader dataset applications. Secondly, the pivotal role of big data technologies lies in facilitating streamlined data storage and rapid data access, crucial for real-time monitoring of user health status and prompt feedback provision. Additionally, big data analytics technologies significantly bolster the training and optimization processes of machine learning models, thereby enhancing their generalization capabilities and prediction accuracy. Specifically, the Spark big data processing platform is employed, chosen for its adeptness in data computation and its robust support for model training and analysis through its integrated machine learning library (MLlib). While the current dataset size may somewhat curtail the full potential of big data technology, there is a firm belief that as data volume grows and data sources diversify, big data technology will assume an increasingly pivotal role in the research and development of smart fitness systems. To mitigate the constraint posed by dataset size, future research avenues may explore data augmentation techniques to enrich dataset diversity, thereby enhancing model robustness. Moreover, meticulous optimization of machine learning algorithms can ensure sustained exceptional performance and generalization capability on the current limited dataset, better equipping them to tackle data size challenges and deliver more precise and personalized smart fitness services to users. Thus, future research could further improve the dataset by expanding the scope of data collection and increasing the sample size to enhance the accuracy and generalization capability of the system model. Additionally, this work mainly analyzes the design and effects of the aerobics intelligent fitness system but does not conduct in-depth field tests and user surveys on the actual operation of the system. Future research could validate the actual effects and user satisfaction of the system through field experiments and user feedback, thereby providing a more comprehensive assessment of the system's performance and usability. Furthermore, this work does not address the applicability of the aerobics intelligent fitness system to different age groups, different health conditions, as well as issues related to the system's long-term effects and continuous management. Future research could explore these aspects to investigate the system's applicability and effectiveness in a broader population and how to maintain health effects through long-term monitoring and management.

In summary, future research could focus on algorithm optimization, data enhancement, field validation, user satisfaction, and long-term effects to further enhance the performance and practicality of the aerobics intelligent fitness system, providing more effective support and assistance for people's healthy lifestyles.

## CRediT authorship contribution statement

**Yuanxin Liu:** Writing – original draft, Visualization, Validation, Supervision, Software, Resources, Methodology, Investigation, Formal analysis, Data curation, Conceptualization. **Shufang Cao:** Writing – review & editing, Visualization, Validation, Supervision, Software, Resources, Methodology.

## Declaration of competing interest

The authors declare that they have no known competing financial interests or personal relationships that could have appeared to influence the work reported in this paper.
